# Periodontal Microbiological Status Influences the Occurrence of Cyclosporine-A and Tacrolimus-Induced Gingival Overgrowth

**DOI:** 10.3390/antibiotics8030124

**Published:** 2019-08-21

**Authors:** Biagio Rapone, Elisabetta Ferrara, Luigi Santacroce, Francesca Cesarano, Marta Arazzi, Lorenzo Di Liberato, Salvatore Scacco, Roberta Grassi, Felice Roberto Grassi, Antonio Gnoni, Gianna Maria Nardi

**Affiliations:** 1 Department of Basic Medical Sciences, Neurosciences and Sense Organs, “Aldo Moro” University of Bari, 70122 Bari, Italy; 2 Complex Operative Unit of Nephrology and Dialysis, Hospital S.S. Annunziata, 66100 Chieti, Italy; 3 Ionian Department (DJSGEM), “Aldo Moro” University of Bari, 70122 Bari, Italy; 4 Department of Dental and Maxillofacial Sciences, “Sapienza” University of Rome, 00100 Rome, Italy; 5 Department of Biomedical Sciences, University of Sassari, 07100 Sassari, Italy

**Keywords:** periodontitis, oral pathogens, periodontal inflammation, drug-induced gingival overgrowth (DIGO), immunosuppressant, cyclosporine-A, Tacrolimus calcium channel blockers, sepsis, kidney transplantation

## Abstract

Immune suppressed renal transplant patients are more prone to developing oral tissue alterations due to medications associated with a pleiotropic set of side effects involving the oral cavity. Drug-induced gingival overgrowth (DIGO) is the most commonly encountered side effect resulting from administration of calcineurin inhibitors such as cyclosporine-A (CsA), the standard first-line treatment for graft rejection prevention in transplant patients. Pathogenesis of gingival overgrowth (GO) is determined by the interrelation between medications and a pre-existing inflammatory periodontal condition, the main modifiable risk factor. Severity of gingival hyperplasia clinical manifestation is also related to calcium channel blocker association, frequently provided in addition to pharmacological therapy of transplant recipients. Specifically, nifedipine-induced enlargements have a higher prevalence rate compared to amlodipine-induced enlargements; 47.8% and 3.3% respectively. Available epidemiological data show a gender difference in prevalence, whereby males are generally more frequently affected than females. The impact of GO on the well-being of an individual is significant, often leading to complications related to masticatory function and phonation, a side effect that may necessitate switching to the tacrolimus drug that, under a similar regimen, is associated with a low incidence of gingival lesion. Early detection and management of GO is imperative to allow patients to continue life-prolonging therapy with minimal morbidity. The purpose of this study was threefold: firstly, to determine the prevalence and incidence of GO under the administration of CsA and Tacrolimus; secondly, to assess the correlation between periodontal status before and after periodontal therapy and medications on progression or recurrence of DIGO; and finally, to analyse the effect of immunosuppressant in association to the channel blocker agents on the onset and progression of gingival enlargement. We compared seventy-two renal transplant patients, including 33 patients who were receiving CsA, of which 25% were also receiving nifedipine and 9.72% also receiving amlodipine, and 39 patients who were receiving tacrolimus, of which 37.5% were also receiving nifedipine and 5.55% also receiving amlodipine, aged between 35 and 60 years. Medical and pharmacological data were recorded for all patients. Clinical periodontal examination, in order to establish the inflammatory status and degree of gingival enlargement, was performed at baseline (T0), 3 months (T1), 6 months (T2), and 9 months (T3). All patients were subjected to periodontal treatment. Statistically significant correlation between the reduction of the mean value of periodontal indices and degree of gingival hyperplasia at the three times was revealed. The prevalence of GO in patients taking cyclosporine was higher (33.3%) in comparison with those taking tacrolimus (14.7%). In accordance with previous studies, this trial highlighted the clinical significance of the pathological substrate on stimulating drug-induced gingival lesion, confirming the key role of periodontal inflammation in pathogenesis of gingival enlargement, but did not confirm the additional effect of calcium-channel blocker drugs in inducing gingival enlargement.

## 1. Background

Several immunosuppressive agents are associated with many undesired side-effects involving oral tissue alterations [1,2]. Drug-induced gingival overgrowth (DIGO) is the most commonly encountered side effect of a plethora of medications [3], showing a high prevalence in response to calcineurin inhibitors such as cyclosporine-A [4,5,6]. The severity of gingival hyperplasia clinical manifestation is also related to calcium channel blocker agent association, frequently provided in addition to pharmacological therapy for transplant recipients [7,8]. Specifically, the nifedipine-induced enlargements have a higher prevalence rate compared to the amlodipine-induced enlargements, that is, 47.8% and 3.3% respectively [9,10]. According to epidemiological available data, although reports vary widely, the overall estimated prevalence is about 30% with a range from 6% to 81% [2], but the occurrence has not been systematically compiled. A higher prevalence (from 48% to 60%) has been reported in the case of therapy combining cyclosporine and nifedipine or amlodipine therapy [11,12]. Males are more commonly affected than females by a ratio of 3:1 [13]. The underlying pathophysiology of gingival enlargement is multifactorial and may vary considerably between individuals [14,15]. One factor believed to play a critical role in the pathogenesis of the lesion is bacterial plaque accumulation and pre-existing periodontal inflammatory status [16,17]. Furthermore, the exposure to bacterial plaque and poor oral hygiene are well-established amplifier risk factors for severity degree of DIGO. In addition, there is some evidence concerning the contribution of genetic factors to the risk of the onset of GO [18]. However, the specific mechanism responsible for the variability is still unclear.

Clinically, hyperplasia first frequently appears within 1–3 months following initiation of immunosuppressant therapy, involving primarily the interproximal gingival area. Gradually, fibrotic enlargement evolves, extending up to cover dental crowns [19,20]. Histologically, it is described as connective tissue disorder, characterized by excessive interstitial collagen deposition, due to metabolic alteration of gingival fibroblasts, increased inflammatory response and altered vascularization. It’s widely recognized that connective tissue fibroblasts demonstrate heterogeneity in response to various stimuli [21,22]. In addition to the commonly seen histopathological patterns of lesions, consisting of an accumulation of connective tissue matrix within the gingival propria, distinct and phenotypically stable subpopulations of fibroblasts existing within gingiva and other connective tissues characterize the damaged tissue [23]. Altered production of collagen and of the other components of the extracellular matrix, the imbalance between synthesis and degradation of the ECM proteins and an altered proliferation rate typify these subpopulations functionally [23]. In addition, environmental factors are involved in disease predisposition [24,25,26]. Histological studies suggest that susceptibility is genetically determined in the cyclosporine-sensitive subpopulation of gingival fibroblasts [27]. Additional evidence supporting fibroblast heterogeneity has been provided by laboratory animal studies, showing an increase of gingival fibroblast growth and synthetic activity by cyclosporine-A [28]. Cell-to-cell variability in the population growth rate has since been observed actively [29,30,31,32]. Interestingly, in order to assess fibroblast heterogeneity in collagenolytic response to cyclosporine collagenases activity, and tissue inhibitor of metalloproteinase (TIMP) in 14 different strains of connective tissue fibroblasts obtained from healthy individuals without periodontal inflammation were studied in vitro [33,34]. The results confirmed the influence of interindividual susceptibility to CsA-induced gingival overgrowth. Some studies reported a wide inter-individual variability [35,36] depending primarily on hereditary genetic variation [37] in drug metabolizing enzymes as a consequence of cytochrome P450 genetic polymorphisms [21,37,38], a major source of variability of response to drug pharmacokinetics. Specifically, cyclosporine is metabolized by CYP3A4 cytochrome P450 isoenzyme [39,40,41]. However, cytochrome P450 is also altered in common clinical conditions (e.g., diabetes mellitus) usually associated with periodontal infections [42,43,44], sometimes evolving in harmful conditions in immune suppressed patients [45,46,47].

The management options for gingival DIGO includes a customized oral hygiene protocol, known as dental biofilm detection topographic technique (D-Biotech) [48], which facilitates ergonomic and non-invasive treatment, and non-surgical periodontal therapy to reduce the inflammatory component, and a consequent surgical approach is needed for reduction of gingival mass. [48].

## 2. Materials and Methods

Our study analyzed data from 72 kidney transplant patients, from the Complex Nephrology Operative Unit, S.S. Annunziata of Chieti. Written informed consent was obtained from all patients in a pre-operative clinic assessment. Patients underwent a comprehensive periodontal examination including plaque index (PI), gingival index (GI), pocket depth (PD), clinical attack loss (CAL). The measurements were determined at six sites per tooth (mesiobuccal, midbuccal, distobuccal, mesiolingual, midlingual, and distolingual), excluding third molars. The Miller and Damm (1992) modified index for enhanced assessment of gingival overgrowth was used. The vertical gingival overgrowth index was described as: (a) Grade 0: normal gingival, no alteration; (b) Grade 1: minimal overgrowth, ≤2 mm, gingiva covering the cervical third or less of the anatomic crown; (c) Grade 2: moderate overgrowth: 2 to 4 mm, gingival covering the middle third of the anatomic crown; (d) Grade 3: severe overgrowth: ≥4 mm, nodular growth, gingival covering more than two thirds of the dental crown. The horizontal gingival overgrowth index is described as: (a) Grade 0: <1 mm; (b) Grade 1: 1 to 2 mm; (c) Grade 2: >2 mm. The possible onset of gingival overgrowth was also recorded, with the related degree of severity (from 0 to 3). The clinical examination was performed by a single calibrated examiner using a manual probe (UNC-15, Hu-Friedy Manufacturing Company, Inc., Chicago, IL, USA). Periodontal treatment was performed using ultrasonic and manual instruments. Clinical evaluations were assessed at baseline and at 3 months, 6 months and 9 months.

## 3. Statistical Analysis

All data obtained during the study were recorded in individual charts and then transferred to the database in Excel Office 2013. The data were processed and analyzed using Statistical Package for the Social Sciences (IBM SPSS 19.0, SPSS Inc., Chicago, IL, USA) version 19. Descriptive statistical analysis included the arithmetic mean and standard deviation. Drug-induced gingival overgrowth prevalence and incidence were calculated with the direct method. The Mann-Whitney test was used to compare medians between treatments. Non-parametric test (Kruskall-Wallis H test) was used to calculate the difference in medians between two independent samples (immunosuppressive treatment). Pearson’s correlation was used to assess the relationship between prevalence of DIGO and periodontal clinical parameters at different times: T0 (baseline), T1 (three months), T2 (six months), T3 (nine months).

**Figure 1:**
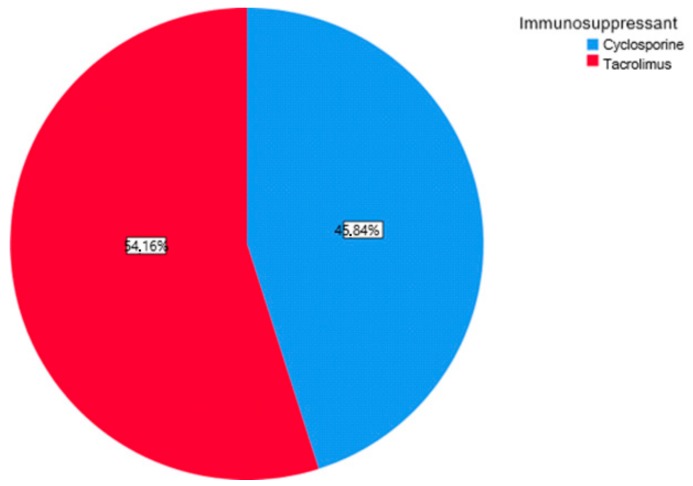
Percentage distribution of immunosuppressive treatments.

**Figure 2:**
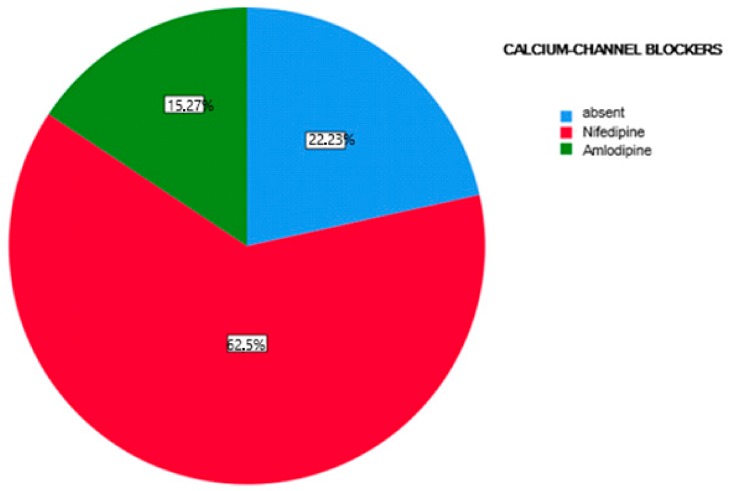
Percentage distribution of additional treatment with calcium channel blockers.

**Figure 3:**
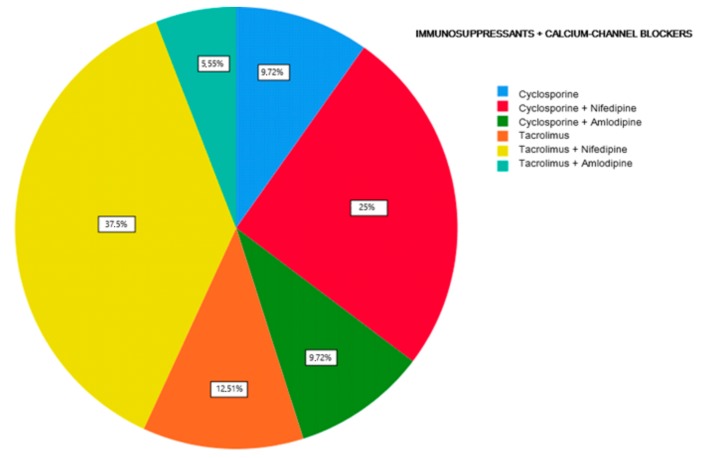
Percentage distribution of immunosuppressive therapies + calcium-channel blockers.

The first part of the study saw a descriptive analysis of the population in question (Figure 1, Figure 2 and Figure 3).

## 4. Results

**Figure 4:**
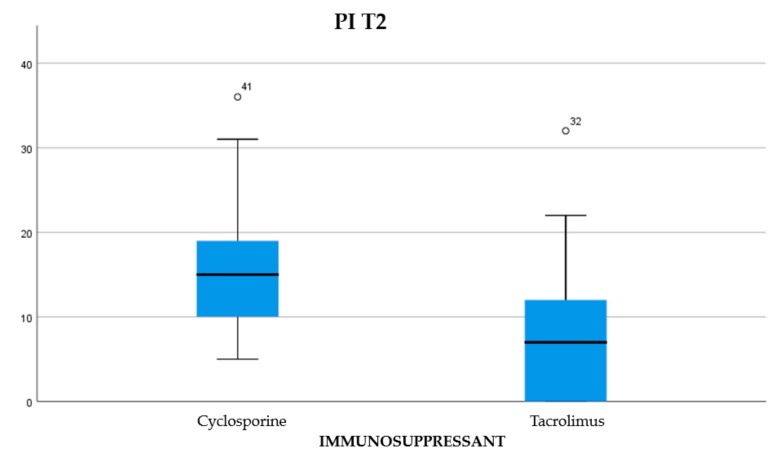
The difference between the medians for PI at T2.

**Figure 5:**
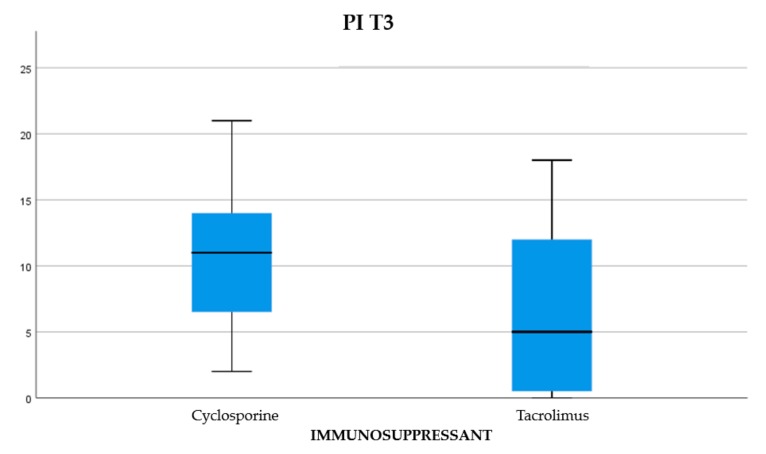
The difference between the medians for plaque index (PI) at T3.

**Figure 6:**
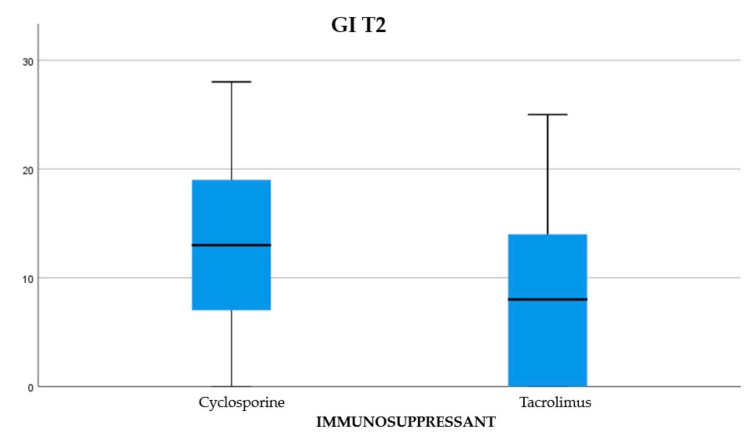
The difference between the medians for gingival index (GI) at T2.

**Figure 7:**
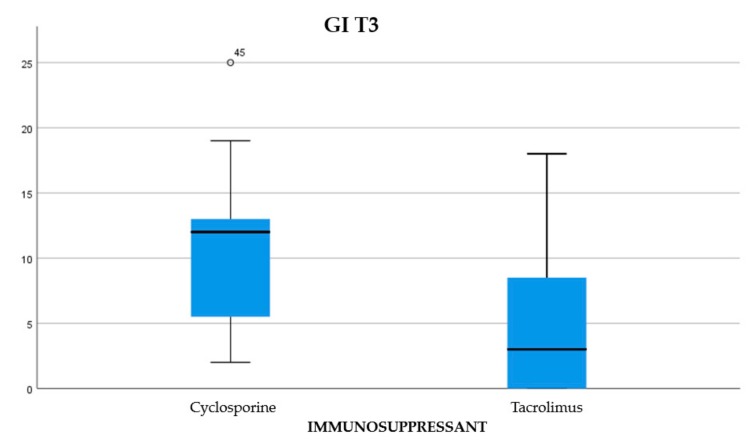
The difference between the medians for GI at T3.

**Figure 8:**
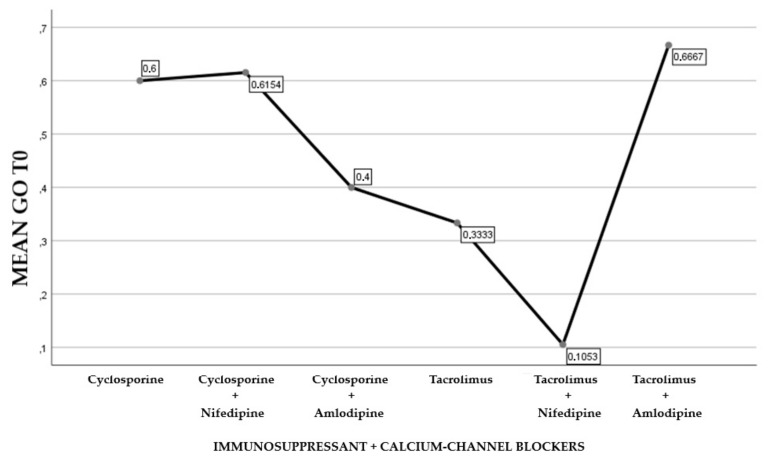
Gingival overgrowth mean resulting by the combination of immunosuppressants and calcium-channel blockers at T0.

**Figure 9:**
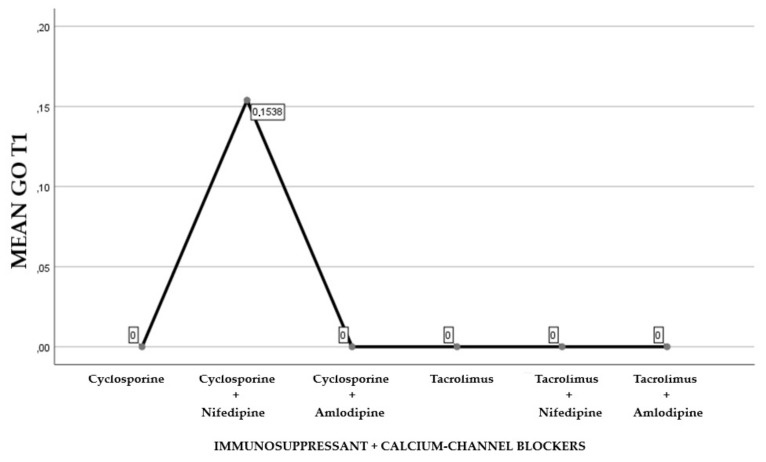
Gingival overgrowth mean with the combination of immunosuppressants and calcium-channel blockers at T1.

**Figure 10:**
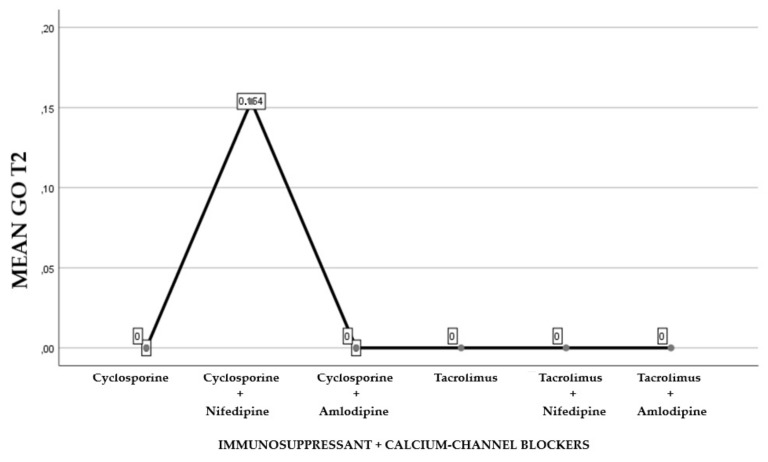
Gingival overgrowth mean with the combination of immunosuppressants and calcium-channel blockers at T2.

**Figure 11:**
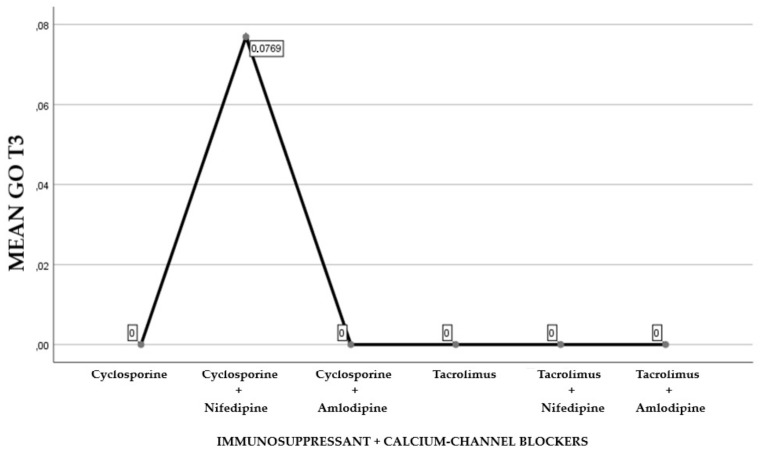
Gingival overgrowth mean resulting by the combination of immunosuppressants and calcium-channel blockers at T3.

About 45.84% of the participants were treated with cyclosporine and 54.16% reporting treatment with tacrolimus (Graph 1). Of the examined population, 22.23% received immunosuppressant therapy without additional calcium-channel blockers, 62.5% received immunosuppressant drug administration in addition to the nifedipine, and 15.27% with the amlodipine (Graph 2). The clinical parameter GO showed no differences between cyclosporine and tacrolimus groups at baseline, 3, 6, and months; PD and CAL also showed no significant differences between groups at 4 times; and the tacrolimus group showed significantly greater GI and PI reduction at 3, 6 and 9 months than the cyclosporine group. Significant correlation between PI and GI was observed (*P* < 0.05) (Table 1). The difference in the medians of GI and PI of the two groups at 6 and 9 months is less in the tacrolimus group than in the cyclosporine group (Figure 4, Figure 5, Figure 6 and Figure 7). When conducting an intergroup comparison, no difference was found in the medians for immunosuppressant treatments in addition to calcium-antagonists and periodontal parameters at, 3, 6 and 9 months follow-up (Figure 8, Figure 9, Figure 10 and Figure 11). The decrease in the GO parameter was more in the tacrolimus plus calcium-channel blocker group compared with the other treatment groups, but the difference was not statistically significant (*P* > 0.05) (Table 2 and Table 3). Results of a correlation analysis showed a positive correlation of GO with all variables (*P* < 0.05), but with exceptions as follows: at 3 months, except PD; at 6 months except, PI and GI; at 9 months except PI and GI (Table 4, Table 5, Table 6 and Table 7).

## 5. Discussion

The current state of knowledge of the prevalence of drug-induced gingival overgrowth is derived from retrospective studies and clinical trials with high heterogeneity in method. The estimates of cumulative prevalence of DIGO in transplant patients treated by immunosuppressant ranges from 6% to 81% [3]. It has been reported that gingival lesion occurs mainly in patients with poor oral health and compromised periodontal status [31]. Therefore, in the present study, gingival overgrowth was strictly analyzed previously as a lesion linked to pre-existent periodontal inflammation. Then, the effect of varying immunosuppressants was evaluated in combination with calcium-channel blockers (CCBs), demonstrating no statistical differences in degree of gingival enlargement between groups. Our findings are consistent with previous studies which have also confirmed that patients in treatment with cyclosporine exhibit significantly greater risk of gingival overgrowth onset compared with patients in treatment with tacrolimus [22,23,24].

The specific mechanism of cyclosporine-induced gingival overgrowth is uncertain. One explanation may be that the accumulation of gingival fibroblasts may result from the inhibition of apoptosis [49], because of the direct effect of cyclosporine metabolites on gingival fibroblasts, protein synthesis and collagen production [50]. Further, in vitro studies were conducted to investigate the pro-proliferative, antiproliferative [51], pro-apoptotic, antiapoptotic [52], or no effect of cyclosporine on gingival keratinocytes, reporting an increased release of keratinocyte growth factor in epithelial cells observed in CsA-induced gingival hyperplasia, but the findings were inconclusive.

The results of the present study are also in accordance with previous studies showing that the degree of gingival overgrowth may favorably improve after the periodontal inflammation clinical parameters decrease. Indeed, it has been amply demonstrated that the upregulation of salivary concentrations of proinflammatory cytokines such as interleukin (IL) 1α, IL-8, and IL-6 is implicated in the pathogenesis of cyclosporine-induced gingival overgrowth. So, maintaining periodontal health may markedly reduce the severity of CsA-induced gingival hyperplasia [52,53].

## 6. Conclusions

Drug-induced gingival overgrowth represents the most frequent adverse reaction in patients taking immunosuppressant drugs, with lower incidence in patients taking tacrolimus. In addition, many studies have reported a higher prevalence of gingival lesion in patients also receiving calcium channel blockers. In our study, a significant positive correlation was found between prevalence of gingival overgrowth and cyclosporine. The slightly lesser GO degree in the calcium-channel blocker group compared to the cyclosporine group may be attributed to the low dosage of drugs.

Although the disorder recognizes a multifactorial genesis, periodontal status is critical in determining the severity and recurrence of the lesion. In conclusion, the results of the present study showed a significant improvement in clinical parameters such as GI, PI and GO between groups, confirming the key role of periodontal inflammation in the pathogenesis of gingival enlargement.

## Figures and Tables

**Table 1:** Kruskall-Wallis (non-parametric) test for median difference between two independent samples (immunosuppressive treatment).

Statistic Tests ^a^				
Periodontal Indices	U of Mann-Whitney	W of Wilcoxon	Z	Asymptotic Sign. (with Two Tails)
PI T0	269,500	675,500	−994	0.320
PI T1	269,500	675,500	−996	0.319
PI T2	165,500	571,500	−2974	0.003
PI T3	189,000	595,000	−2528	0.011
GI T0	230,000	636,000	−1742	0.081
GI T1	235,000	641,000	−1649	0.099
GIT2	184,500	590,500	−2616	0.009
GI T3	133,000	539,000	−3597	0.000
GO T0	250,000	656,000	−1744	0.081
GO T1	308,000	714,000	−1103	0.270
GO T2	308,000	714,000	−1103	0.270
GO T3	308,000	714,000	−1103	0.270
PD T0	279,500	685,500	−805	0.421
PD T1	278,500	684,500	−824	0.410
PD T2	283,000	689,000	−739	0.460
PD T3	299,500	705,500	−428	0.669
CAL T0	278,500	684,500	−824	0.410
CAL T1	272,000	678,000	−947	0.343
CAL T2	253,000	659,000	−1307	0.191
CAL T3	259,000	665,000	−1194	0.232

^a.^ Grouping variable: Immunosuppressants.

**Table 2:** Kruskall-Wallis H test for the difference between GO means.

Statistic Tests ^a,b^				
Test	GO T0	GO T1	GO T2	GO T3
H of Kruskal-Wallis	6434	2923	2923	2923
gl	5	5	5	5
Asymptotic sign.	0.266	0.712	0.712	0.712

^a.^ Kruskal Wallis’s Test; ^b.^ Grouping variable: Immunosuppressants + calcium-channel blockers.

**Table 3:** Correlations between GO and periodontal indices.

Statistics Tests ^a,b^			
Periodontal Indices	H of Kruskal-Wallis	Gl	Asymptotic Sign.
PI T0	7880	5	0.163
PI T1	5710	5	0.335
PI T2	11,280	5	0.046
PI T3	6776	5	0.238
GI T0	7979	5	0.157
GI T1	6311	5	0.277
GI T2	9126	5	0.104
GI T3	13,809	5	0.017
PD T0	7601	5	0.180
PD T1	6852	5	0.232
PD T2	7661	5	0.176
PD T3	7136	5	0.211
CAL T0	6757	5	0.239
CAL T1	3897	5	0.564
CAL T2	5310	5	0.379
CAL T3	6844	5	0.232

^a.^ Kruskal Wallis Test; ^b.^ Grouping variable: Immunosuppressants + calcium-channel blockers.

**Table 4:** Correlation between GO degree and periodontal indices at baseline.

Correlations		
		IG T0
GO T0	Pearson’s correlation	1
	N	51
PI T0	Pearson’s correlation	0.512
	Sign. (with two tails)	0.000
	N	51
GI T0	Pearson’s correlation	0.621
	Sign. (with two tails)	0.000
	N	51
PD T0	Pearson’s correlation	0.351
	Sign. (with two tails)	0.012
	N	51
CAL T0	Pearson’s correlation	0.358
	Sign. (with two tails)	0.010
	N	51

**Table 5:** Correlation between GO degree and periodontal indices at T1.

Correlations		
		IG T1
GO T1	Pearson’s correlation	1
	N	51
PI T1	Pearson’s correlation	0.625
	Sign. (with two tails)	0.000
	N	51
GI T1	Pearson’s correlation	0.455
	Sign. (with two tails)	0.001
	N	51
PD T1	Pearson’s correlation	0.243
	Sign. (with two tails)	0.085
	N	51
CAL T1	Pearson’s correlation	0.333
	Sign. (with two tails)	0.017
	N	51

**Table 6:** Correlation between GO degree and periodontal indices at T2.

Correlations		
		IG T2
GO T2	Pearson’s correlation	1
	N	51
PI T2	Pearson’s correlation	0.183
	Sign. (with two tails)	0.200
	N	51
GI T2	Pearson’s correlation	0.163
	Sign. (with two tails)	0.253
	N	51
PD T2	Pearson’s correlation	0.303
	Sign. (with two tails)	0.031
	N	51
CAL T2	Pearson’s correlation	0.383
	Sign. (with two tails)	0.006
	N	51

**Table 7:** Correlation between GO degree and periodontal indices at T3.

Correlations		
		IG T3
GO T3	Pearson’s correlation	1
	N	51
PI T3	Pearson’s correlation	0.183
	Sign. (with two tails)	0.200
	N	51
GI T3	Pearson’s correlation	0.163
	Sign. (with two tails)	0.253
	N	51
PD T3	Pearson’s correlation	0.303
	Sign. (with two tails)	0.031
	N	51
CAL T3	Pearson’s correlation	0.383
	Sign. (with two tails)	0.006
	N	51

## Finding

This research received no external funding.
